# The establishment of neuron-specific enolase reference interval for the healthy population in southwest China

**DOI:** 10.1038/s41598-020-63331-x

**Published:** 2020-04-14

**Authors:** Qiang Miao, Bei Cai, Xuedan Gao, Zhenzhen Su, Junlong Zhang

**Affiliations:** Department of Laboratory Medicine/ Research Center of Clinical Laboratory Medicine, West China Hospital of Sichuan University, Chengdu, China

**Keywords:** Lung cancer, Tumour biomarkers, Biomarkers, Diseases, Medical research

## Abstract

To investigate and establish a reference interval (RI) of neuron-specific enolase (NSE) in southwest China’s healthy population by using the laboratory information system database. A total of 86957 periodic health examination individuals of the medical examination center in West China Hospital from 2016 to 2018 were included in the study. We used the Box-Cox conversion combined with the Tukey method to normalize the data and eliminate the outliers, and the normal distribution method and the nonparametric method to estimate the 95% distribution RI. The NSE 95% distribution RI we established in healthy populations in southwest China through normal distribution and nonparametric method were 0–19.64 ng/ml and 0–20.46 ng/ml, respectively. The obtained RIs verification conformed to the standard and was significantly different from the reagent instruction(*P* < 0.05). The RI established by the nonparametric method was superior to the RI of the normal distribution method and reagent instruction(*P* < 0.05). We initially established an NSE RI that was suitable for the healthy southwest China population. The Box-Cox conversion combined with the Tukey method and nonparametric method is a reliable and straightforward indirect method for reference interval acquisition, which is suitable for the promotion and application of clinical laboratory.

## Introduction

Neuron-specific enolase (NSE) is an acidic protease unique to neurons and neuroendocrine cells. It is a sensitive indicator for assessing the severity of nerve cell damage and prognosis^1,2^. It is also specific markers for tumors such as neuroblastoma and small cell lung cancer (SCLC)^3,4^. Different detection methods have different reference intervals (RI). With the application of NSE in nervous system diseases and tumors more and more widely, appropriate RI of NSE is essential for a health evaluation, cancer diagnosis, therapy monitoring, and prognosis assessment.

The RI is one of the critical basis for clinicians to judge whether the test result is healthy or not and to make clinical decisions^5^. At present, there are few studies on NSE RI in our country^6–8^, and most hospitals adopt the reference range provided by the reagent specification as the standard and ignoring the existence of differences in different countries, regions, ages, and gender. The documents CLSI EP28-A3C^9^ published by The Clinical and Laboratory Standards Institute encourage every laboratory to establish its own RI. Although a few studies from China have reported the RI of NSE^6–8^, there have not been reported from the southwest China population so far. Our laboratory currently uses the RI recommended by the reagent manual. In practice, we found that it does not meet the southwest China population. Therefore, it is necessary to establish the RI of NSE for people in southwest China.

The indirect method which establishing RI based on mathematical-statistical models using data already available in a laboratory database^10–12^ has been approved by the CSLI EP28-A3C^9^. In this study, we used the indirect method to establish the RI of NSE for the southwest China population from 86957 cases of periodic health examination data in the Laboratory Information System (LIS) of West China Hospital from 2016 to 2018. Which provides a basis for clinical correct diagnosis and treatment.

## Materials and methods

### Subjects

We collected 93,398 cases NSE test data from periodic health examination individuals in physical examination centers of West China Hospital from 2016 to 2018. We excluded 2,245 cases of duplicate individuals according to physical examination number, name, and age. We excluded 1,250 cases of NSE test results with hemolysis remarks and 2,478 individuals with abnormal blood routine indicators and liver and kidney function indicators. We further excluded 468 individuals with a history of tumor disease, neurological disease, some benign diseases, hypertension, diabetes, pregnancy and lactation, recent surgery and hospitalization through a physical examination report. Through the above executable and straightforward exclusion criteria, we finally included the NSE test data of 86,957 healthy individuals who did not show significant abnormalities in the results of the physical examination. There are 50,210 males and 36,747 females with an age range of 14–100 years. This study protocol was approved by the Ethics Committee of West China Hospital of Sichuan University and was performed in accordance with the Declaration of Helsinki. All involved participants provided written informed consent.

### Instruments and reagents

The serum NSE was quantitatively measured by Electrochemiluminescence assay (ECLIA) on a Roche Cobas e601 fully automated immunoassay system (Roche Diagnostics GmbH, Germany). Roche Diagnostics GmbH provided the detection kits and calibrators. The quality control adopts the Lyphochek Tumor Marker Plus control products from Bio-Rad (USA). Our laboratory uses Westgard multi-rules (1_3 S_, 2_2 S_, R_4S_) for internal quality control. The cumulative coefficient of variation (CV) at both high and low levels was less than 8%. The test project participated in the quality evaluation activities organized by the clinical laboratory center of the National Health Commission and the College of American Pathologists (CAP) every year. The results were satisfactory, and the offset was acceptable.

### RI establishment methods

First of all, we used the Skewness-Kurtness test to analyze the normality of the data. When the skewness value and the kurtosis value are less than 1.96 times the standard deviation of the corresponding skewness and kurtosis, respectively, it can judge as a normal distribution. The non-normally distributed data is transformed into approximately normal distribution by the Box-Cox transformation. Secondly, we used the Tukey method to eliminate outliers^13,14^. The upper limit is P_75_ plus 1.5 times the interquartile range (IQR), and the lower limit is P_25_ minus 1.5 times the IQR. All values outside this range should be considered outliers. Then all the subjects were grouped into subgroups according to gender (male and female) and age(14–17, 18–30, 31–45, 46–60, 61–75, 76–90, and 91–100 years which referred to the world health organization’s (WHO) age division standard and the traditional Chinese age division standard). The ANVOA analysis was used to evaluate whether age and gender were possible grouping factors. The standard normal deviate test (z-test) suggested by Harris and Boyd was used to assess whether or not to partition RIs by the subclass. Finally, the normal distribution method and the nonparametric method were used to estimate the 95% distribution RI of NSE. For NSE, only the high values are of clinical concern. Therefore we used the one-side RI. The upper reference limit defined as 95th percentiles of the distribution and mean+1.645 SD for nonparametric and normal distribution methods, respectively. All arrangements were referenced to the documents CLSI EP28-A3C^9^ published by The Clinical and Laboratory Standards Institute.

### Reference interval verification

According to the screening conditions of this experiment, 10765 cases of apparently healthy people who examined in our physical examination center from July to August 2019 were used for the validation of the obtained RIs. Calculating the ratio of individuals outside the RI. If the rate was less than 5%, the RI was verified^9^.

### Statistical analysis

The data were analyzed by SPSS version 19.0 for Windows (SPSS Inc., Chicago, IL, USA). Quantitative variables were described as mean ± SD or percentiles. Continuous data were checked for normal distribution by the Skewness-Kurtness test. The ANVOA analysis and z-test were used to determine whether to divide the RIs according to the subclass. Using Stata 15.0 statistical software performs Box-Cox normality transformation of data (undetermined parameters λ are obtained by maximum likelihood method). The Chi-square test was used to compare the differences in rates and composition ratios. A value of *P* < 0.05 was considered statistically significant.

## Results

### Distribution of data and elimination of outliers

The skewness and kurtosis of NSE data obtained from LIS were 4.355 and 54.84, respectively, which is non-normally distributed by the Skewness-Kurtness test. After Box-Cox conversion, the skewness and kurtosis of NSE data were 0.209 and 1.27, showing an approximately normal distribution. The results before and after the transformation are shown in Table 1. We used the Tukey method to eliminate 1842 cases of outliers (male 1126, female 716) and obtained a total of 85,115 reference individuals (male 49084, female 36031). See Table 2 for detailed parameters before and after elimination.

**Table 1:** NSE data normality test and BOX-COX conversion results.

N	λ	Before Conversion				After Conversion			
		Mean	SD	Skewness	Kurtosis	Mean	SD	Skewness	Kurtosis
86957	−0.467	14.58	4.37	4.355	54.84	1.51	0.07	0.209	1.27

**Table 2:** The data before and after outliers were eliminated by the Tukey method.

Gender	Before eliminate						After eliminate					
	N	P_25_	P_75_	IQR	Max	Min	N	P_25_	P_75_	IQR	Max	Min
Male	50210	12.38	16.7	4.32	143.5	4.08	49084	12.38	16.54	4.16	27.57	8.24
Female	36747	11.58	15.36	3.78	121.2	4.55	36031	11.62	15.32	3.7	27.56	8.24

### Establishment of the reference interval

We grouped the data by gender and age. The levels of NSE were significantly different among age and gender groups. The results of ANVOA analysis indicated that age and gender might be the dividing factors of NSE RI (p < 0.05, Supplementary material Table S1). But, further Z test results showed that it is not necessary to set gender- and age-specific RI by subclass (Supplementary material Tables S2 and S3). The 95% distribution RI upper limit of NSE after excluding outliers were calculated by the normal distribution method and the nonparametric method, respectively. For reference populations aged 14–100 years included in the study. The NSE RI estimated by the normal distribution method was 0–19.64 ng/ml (14–100 years, the 90% confidence interval was 19.62–19.66 ng/ml). Using the nonparametric method to estimate the NSE RI was 0–20.46 ng/ml (the 90% confidence interval was 20.4–20.54 ng/ml). All results were shown in Table 3.

**Table 3:** The NSE reference interval calculate by parametric and nonparametric methods and the verifying results.

Parameter	reference population	Verify population
Age (range, year)	14–100	14–90
Gender (M/F)	49084/36031	5851/4914
Mean+1.645 SD (ng/ml)	19.64	—
P95 (ng/ml)	20.46	—
RI^1^(90% CI, ng/ml)	0–20.46 (20.4–20.54)	—
RI^2^(90% CI, ng/ml)	0–19.64 (19.62–19.66)	—
RI^3^(ng/ml)	16.3	—
N1(%)	—	401 (3.7)
N2(%)	—	504 (4.7)
N3(%)	—	1701 (15.8)

RI^1^: reference interval defined by nonparametric method (P95); RI^2^: reference interval determined by the traditional parameter method (mean+1.645 SD); RI^3^: reference interval from reagent specification.

N1: the numbers of verify population individuals outside the RI^1^; N2: the numbers of verify population individuals outside the RI^2^; N3: the numbers of verify population individuals outside the RI^3^;

### Verification and comparison of reference intervals

The ratios of individuals outside the RI estimated by this research in Table 3 were all less than 5%. More than 95% apparently healthy individuals’ NSE levels were within the RI, so the verification passed. Among the verify population, 401(3.7%) cases were diagnosed as abnormal according to the RI^1^ established by the nonparametric method in this study. 504(4.68%) individuals were diagnosed as abnormal with the RI^2^ estimated by the traditional parameter method. However, there were 1701(15.8%) cases diagnosed as abnormal according to the RI^3^ of the reagent specification. There was a statistically significant difference in the ratio of positive judgments between three different reference intervals in the verification population (*P* < 0.05). The pairwise comparison is also statistically significantly different; the positive rate of RI^1^ is markedly lower than that of RI^2^ and RI^3^ (Table 4).

**Table 4:** The comparison results of verify population by different NSE reference intervals.

	RI^1^(0–20.46 ng/ml)	RI^2^(0–19.64 ng/ml)	RI^3^(0–16.3 ng/ml)	*P*
Negative(within the RI)	10364 (96.3%)^**a**^	10261 (95.3%)^**b**^	9064 (84.2%)^**c**^	0.000*
Positive (outside the RI)	401 (3.7%)^**a**^	504 (4.7%)^**b**^	1701 (15.8%)^**c**^	
Tatal	10765	10765	10765	

*The three reference intervals were statistically significantly different.

a, b, c: the following pairwise comparison are statistically significantly diffreent.

## Discussion

NSE is a glycolytic enzyme present in the cytoplasm of neurons cells, which is found in high concentrations in brain tissue. NSE is also present in small amounts in erythrocytes, platelets, and plasma, which explains its lower physiological levels^15^. At present, NSE is widely used as a judgment indicator for the severity of brain tissue damage^1,2^. Besides, it is overexpressed in tumors associated with the origin of neuroendocrine tissues, particularly SCLC. As a sensitive and specific tumor marker for SCLC, it can be used for differential diagnosis and monitoring the therapeutic effect of radiotherapy and chemotherapy for SCLC^3,16^. Therefore, the correct assessment of serum NSE levels has important clinical implications for the early diagnosis, treatment, and prognosis of the disease.

The RI is the standard of making a medical diagnosis, therapeutic assessment, or other physiological assessment. Most clinical decision-making processes are based on information provided by laboratory reports^5^. Therefore, providing reliable RI is an essential task for clinical laboratories^5,9^. At present, the RI used in the clinical laboratory mainly comes from the reagent manufacturer, the National Clinical Laboratory Procedures, other studies. However, due to the influence of race, age, gender, geographical location, and other factors, as well as the detection system, detection methods and reagent sources lead to inconsistent test results. Therefore, the laboratory establishes a local RI that is conducive to the definitive diagnosis and effective treatment of the disease.

The documents CLSI EP28-A3C recommends the direct method to establish the RI. However, as the direct way requires a lot of workforces and material resources to develop the RI, it is challenging to implement under the current laboratory conditions. With the development of information technology, the method of using the indirect method to establish the RI by using a large amount of data stored in the LIS has also been accepted by EP28-A3C and used in many studies^11,17^. This study was based on the large sample of laboratory data from individuals who were undergoing periodic health examinations to establish the NSE RI of the southwest China population through statistical analysis. We created the RI for serum NSE was 0–19.64 (90% CI 19.62–19.66) ng/ml (by normal distribution method) and 0–20.46 (90% CI 20.4–20.54) ng/ml (by nonparametric method)in 14–100 years old apparently healthy southwest China individuals (Table 3). Bjerner *et al*.^18^ reported that the upper 97.5% reference limit of RI for NSE in healthy Nordic individuals was 8.91 ng/ml. Hee-Yeon *et al*.^19^ established the RI of NSE in healthy Korean adults aged 20–60 years was 8.09–16.05 ng/ml for males and 7.24–15.77 ng/ml for females. Due to ethnic differences, It is different from the results of our study. This further illustrates the necessity of establishing RI for our laboratory in the southwest China region. In our study, the upper limit of the RI for male and female in 14–100 years was about 20 ng/ml, which was consistent with the results of JingJing Yang *et al*. and Meihong LU *et al*. reported on the populations of Henan and Jiangsu in China^7,8^. The positive rates determined according to the RI of the reagent specification was 15.8% (Table 4), which will cause overdiagnosis and excessive intervention, causing unnecessary psychological burden and economic burden on the individuals. The RI established by the nonparametric method was superior to the RI of the normal distribution method and reagent instruction through verification and comparison (Table 4), which can better reflect the serum NSE level of healthy individuals in southwest China region and used for clinical reference.

There were several limitations to this study. Firstly, The indirect method used in this study has specific restrictions for the inclusion and exclusion of reference individuals and may include some non-healthy individuals. Secondly, Because of the lack of data on children, we cannot establish the RI of NSE in healthy children.

## Conclusion

In summary, we initially established an NSE RI (0–20.46 ng/ml) that was suitable for the healthy southwest China population, which helped to reduce false positives in brain damage and SCLC diagnosis and avoid misdiagnosis. This study fills the gap in the normal reference interval for NSE in southwestern China. Using the data from individuals who were undergoing periodic health examinations with the Box-Cox conversion combined with the Tukey method and nonparametric method is a reliable and straightforward indirect method for reference interval acquisition, which is suitable for the promotion and application of the clinical laboratory.

## Supplementary information

Supplementary Information 1.

Supplementary Information 2.
