# Supplementary material for: The establishment of neuron-specific enolase reference interval for the healthy population in southwest China
Source: Sci Rep. 2020 Apr 14;10:6332. doi: 10.1038/s41598-020-63331-x (PMC7156405; doi:10.1038/s41598-020-63331-x)
Supplement: Supplementary file 1 — Supplementary Information 1. [file 41598_2020_63331_MOESM1_ESM.docx]

| **Table S1 The ANVOA analysis results of grouping factors age and gender** | | | |
| --- | --- | --- | --- |
| Parameter | N | NSE (ng/ml, mean±SD) | *P* |
| Gender |  |  | 0.000 |
| male | 49084 | 14.74±3.32 |  |
| female | 36031 | 13.75±3.02 |  |
| Age (year) |  |  | 0.000 |
| 14—17 | 48 | 15.78±3.49 |  |
| 18—30 | 11599 | 14.58±3.4 |  |
| 31—45 | 32786 | 14.16±3.26 |  |
| 46—60 | 30935 | 14.3±3.14 |  |
| 61—75 | 8130 | 14.63±3.16 |  |
| 76—90 | 1603 | 14.74±3.32 |  |
| 91—100 | 14 | 13.78±3.71 |  |

The establishment of neuron-specific enolase reference interval for the healthy population in southwest China

Qiang Miao, Bei Cai, Xuedan Gao, Zhenzhen Su, Junlong Zhang ^#^

| **Table S2 The standard normal deviate test results of gender subclass** | | | | |
| --- | --- | --- | --- | --- |
| Gender | N | NSE (ng/ml, mean±SD) | *Z* | *Z** |
| Male | 49084 | 14.74±3.32 | 45.296 | 56.496 |
| Female | 36031 | 13.75±3.02 |  |  |
| $Z=\frac{\vert\bar{X}1-\bar{X}2\vert}{\left[ \left( \frac{{S1}^{2}}{N1} \right)-\left( \frac{{S2}^{2}}{N2} \right) \right]^{\frac{1}{2}}}$ , $Z^{*}=3\left[ \left( N1+N2 \right)/240 \right]^{\frac{1}{2}}$. (*N*1≥120，*N*2≥120)  $\bar{X}1$ and $\bar{X}2$ are the observed means of the two subgroups, S1 and S2 are the observed variances, and *N*1 and *N*2 are the number of reference values in each subclass, respectively. If the calculated Z exceeds Z*, they recommend partitioning. | | | | |

| **Table S3 The results of pairwise comparison of age subclass by standard normal deviate test** | | | | | |
| --- | --- | --- | --- | --- | --- |
| Age groups (year) | | N | NSE (ng/ml, mean±SD) | *Z* | *Z** |
| 18—30 |  | 11599 | 14.58±3.4 |  |  |
|  | 14—17 | 48 | 15.78±3.49 | - | - |
|  | 31—45 | 32786 | 14.16±3.26 | 11.557 | 40.798 |
|  | 46—60 | 30935 | 14.3±3.14 | 7.720 | 39.938 |
|  | 61—75 | 8130 | 14.63±3.16 | 1.060 | 27.200 |
|  | 76—90 | 1603 | 14.74±3.32 | 1.803 | 22.250 |
|  | 91—100 | 14 | 13.78±3.71 | - | - |
| 31—45 |  |  |  |  |  |
|  | 14—17 | 48 | 15.78±3.49 | - | - |
|  | 46—60 | 30935 | 14.3±3.14 | 5.522 | 48.883 |
|  | 61—75 | 8130 | 14.63±3.16 | 11.929 | 39.171 |
|  | 76—90 | 1603 | 14.74±3.32 | 6.835 | 35.911 |
|  | 91—100 | 14 | 13.78±3.71 | - | - |
| 46—60 |  |  |  |  |  |
|  | 14—17 | 48 | 15.78±3.49 | - | - |
|  | 61—75 | 8130 | 14.63±3.16 | 8.390 | 38.275 |
|  | 76—90 | 1603 | 14.74±3.32 | 5.187 | 34.931 |
|  | 91—100 | 14 | 13.78±3.71 | - | - |
| 61—75 |  |  |  |  |  |
|  | 14—17 | 48 | 15.78±3.49 | - | - |
|  | 76—90 | 1603 | 14.74±3.32 | 1.222 | 19.105 |
|  | 91—100 | 14 | 13.78±3.71 | - | - |
| 76—90 |  |  |  |  |  |
|  | 14—17 | 48 | 15.78±3.49 | - | - |
|  | 91—100 | 14 | 13.78±3.71 | - | - |
| $Z=\frac{\vert\bar{X}1-\bar{X}2\vert}{\left[ \left( \frac{{S1}^{2}}{N1} \right)-\left( \frac{{S2}^{2}}{N2} \right) \right]^{\frac{1}{2}}}$ , $Z^{*}=3\left[ \left( N1+N2 \right)/240 \right]^{\frac{1}{2}}$. (*N*1≥120，*N*2≥120)  $\bar{X}1$ and $\bar{X}2$ are the observed means of the two subgroups, S1 and S2 are the observed variances, and *N*1 and *N*2 are the number of reference values in each subclass, respectively. If the calculated Z exceeds Z*, they recommend partitioning. | | | | | |
