# Supplementary material for: The establishment of neuron-specific enolase reference interval for the healthy population in southwest China
Source: Sci Rep. 2020 Apr 14;10:6332. doi: 10.1038/s41598-020-63331-x (PMC7156405; doi:10.1038/s41598-020-63331-x)
Supplement: Supplementary file 2 — Supplementary Information 2. [file 41598_2020_63331_MOESM2_ESM.docx]

@article{NSE,

author = {Bakan, E. and Polat, H. and Ozarda, Y. and Ozturk, N. and Baygutalp, N. K. and Umudum, F. Z. and Bakan, N.},

title = {A reference interval study for common biochemical analytes in Eastern Turkey: a comparison of a reference population with laboratory data mining},

journal = {Biochem Med (Zagreb)},

volume = {26},

number = {2},

pages = {210-23},

note = {Bakan, Ebubekir

Polat, Harun

Ozarda, Yesim

Ozturk, Nurinnisa

Baygutalp, Nurcan Kilic

Umudum, Fatma Zuhal

Bakan, Nuri

Journal Article

Croatia

Biochem Med (Zagreb). 2016;26(2):210-23. doi: 10.11613/BM.2016.023.},

abstract = {INTRODUCTION: The aim of this study was to define the reference intervals (RIs) in a Turkish population living in Northeast Turkey (Erzurum) for 34 analytes using direct and indirect methods. In the present study, the regional RIs obtained were compared with other RI studies, primarily the nationwide study performed in Turkey. MATERIALS AND METHODS: For the direct method, 435 blood samples were collected from a healthy group of females (N = 218) and males (N = 217) aged between 18 and 65 years. The sera were analysed in Ataturk University hospital laboratory using Roche reagents and analysers for 34 analytes. The data from 1,366,948 records were used to calculate the indirect RIs using a modified Bhattacharya method. RESULTS: Significant gender-related differences were observed for 17 analytes. There were also some apparent differences between RIs derived from indirect and direct methods particularly in some analytes (e.g. gamma-glutamyltransferase, creatine kinase, LDL-cholesterol and iron). The RIs derived with the direct method for some, but not all, of the analytes were generally comparable with the RIs reported in the nationwide study and other previous studies in Turkey.There were large differences between RIs derived by the direct method and the expected values shown in the kit insert (e.g. aspartate aminotransferase, total-cholesterol, HDL-cholesterol, and vitamin B12). CONCLUSIONS: These data provide region-specific RIs for 34 analytes determined by the direct and indirect methods. The observed differences in RIs between previous studies could be related to nutritional status and environmental factors.},

keywords = {Adolescent

Adult

Aged

Aspartate Aminotransferases/blood

Blood Chemical Analysis/methods/*standards

Cholesterol, HDL/blood

*Data Mining

Female

Humans

Laboratories, Hospital

Male

Middle Aged

Reference Values

Turkey},

ISSN = {1330-0962 (Print)

1330-0962},

DOI = {10.11613/bm.2016.023},

year = {2016},

type = {Journal Article}

}

@article{NSE,

author = {Bjerner, J. and Hogetveit, A. and Wold Akselberg, K. and Vangsnes, K. and Paus, E. and Bjoro, T. and Bormer, O. P. and Nustad, K.},

title = {Reference intervals for carcinoembryonic antigen (CEA), CA125, MUC1, Alfa-foeto-protein (AFP), neuron-specific enolase (NSE) and CA19.9 from the NORIP study},

journal = {Scand J Clin Lab Invest},

volume = {68},

number = {8},

pages = {703-13},

note = {Bjerner, Johan

Hogetveit, Anita

Wold Akselberg, Katrine

Vangsnes, Kirsti

Paus, Elisabeth

Bjoro, Trine

Bormer, Ole Petter

Nustad, Kjell

eng

England

2008/07/09 09:00

Scand J Clin Lab Invest. 2008;68(8):703-13. doi: 10.1080/00365510802126836.},

abstract = {OBJECTIVE: Adhering to current IFCC recommendations, we calculated upper 97.5 % reference limits for serum tumor markers. MATERIAL AND METHODS: Serum samples from 498 healthy individuals from the Nordic reference interval project (NORIP) were investigated for carcinoembryonic antigen (CEA), CA125 and MUC1 (episialin, CA15.3) using in-house immunofluorometric assays and, for alpha-foetoprotein (AFP), a PerkinElmer Life Sciences assay, neuron-specific enolase (NSE) using an in-house immunoradiometric assay and CA19.9 using a Beckman Access assay. All assays participate in external quality assessment programs. RESULTS: CEA concentrations increased with age and smoking. Upper reference limits for non-smokers were 3.59 microg/L at 50 years and 4.12 microg/L at 70 years. CA125 concentrations were age-independent and the upper reference limit was 35.8 kU/L. MUC1 increased with age and body mass index (BMI). Upper reference limits were 31.7 kU/L at 40 years and BMI 24, 37.5 kU/L at 70 years and BMI 24, and 33.7 kU/L at 40 years and BMI 30. AFP increases with age, and the upper reference limits were 3.82 kU/L at 20 years and 8.70 kU/L at 60 years. An upper reference limit for NSE was 8.91 microg/L in non-smokers; smokers exhibited significantly lower levels. The upper reference limit for individuals expressing CA19.9 was 28.3 kU/L. CONCLUSIONS: For AFP, CA125 and CA19.9, the reference levels obtained were close to previously reported reference ranges. Smoking and age were confirmed as covariates for CEA. The associations between MUC1 with age and BMI and between NSE and smoking have not been reported previously.},

keywords = {Adolescent

Adult

Age Distribution

Aged

Aged, 80 and over

CA-125 Antigen/*blood

CA-19-9 Antigen/*blood

Carcinoembryonic Antigen/*blood

Female

Humans

Male

Middle Aged

Mucin-1/*blood

Phosphopyruvate Hydratase/*blood

Reference Values

Regression Analysis

Scandinavian and Nordic Countries

alpha-Fetoproteins/*analysis},

ISSN = {1502-7686 (Electronic)

0036-5513 (Linking)},

DOI = {10.1080/00365510802126836},

url = {http://www.ncbi.nlm.nih.gov/pubmed/18609108},

year = {2008},

type = {Journal Article}

}

@article{NSE,

author = {CLSI.},

title = {Defining, Establishing, and Verifying Reference Intervals in the Clinical Laboratory; Approved Guideline—Third Edition},

journal = {CLSI document EP28 – A3c ed. Wayne, PA, USA: CLSI (Clinical Laboratory Standards Institute)},

volume = {28},

number = {30},

abstract = {

Clinical and Laboratory Standards Institute document EP28-A3c—Defining, Establishing, and Verifying Reference Intervals in

the Clinical Laboratory; Approved Guideline—Third Edition is written for users of diagnostic laboratory tests. It offers a protocol

for determining reference intervals that meet the minimum requirements for reliability and usefulness. The guideline focuses on

health-associated reference values as they relate to quantitative clinical laboratory tests. Included are various requirements for

studies to determine reference values for a new analyte or a new analytical method of a previously measured analyte. Also

discussed is the transfer of established reference values from one laboratory to another.},

ISSN = {0273-3099},

year = {2010},

type = {Journal Article}

}

@article{NSE,

author = {Concordet, D. and Geffre, A. and Braun, J. P. and Trumel, C.},

title = {A new approach for the determination of reference intervals from hospital-based data},

journal = {Clin Chim Acta},

volume = {405},

number = {1-2},

pages = {43-8},

note = {1873-3492

Concordet, D

Geffre, A

Braun, J P

Trumel, C

Journal Article

Netherlands

Clin Chim Acta. 2009 Jul;405(1-2):43-8. doi: 10.1016/j.cca.2009.03.057. Epub 2009 Apr 5.},

abstract = {BACKGROUND: Reference limits are some of the most widely used tools in the medical decision process. Their determination is long, difficult, and expensive, mainly because of the need to select sufficient numbers of reference individuals according to well-defined criteria. Data from hospitalized patients are, in contrast, numerous and easily available. Even if all the information required for a direct reference interval computation is usually not available, these data contain information that can be exploited to derive at least rough reference intervals. METHODS: In this article, we propose a method for the indirect estimation of reference intervals. It relies on a statistical method which has become a gold-standard in other sciences to separate components of mixtures. It relies on some distributional assumptions that can be checked graphically. For the determination of reference intervals, this new method is intended to separate the healthy and diseased distributions of the measured analyte. We assessed its performance by using simulated data drawn from known distributions and two previously published datasets (from human and veterinary clinical chemistry). RESULTS AND DISCUSSION: The comparison of results obtained by the new method with the theoretical data of the simulation and determination of the reference interval for the datasets was good, thus supporting the application of this method for a rough estimation of reference intervals when the recommended procedure cannot be used.},

keywords = {Animals

Computer Simulation

Creatine/blood

Dogs

*Hospitals

Humans

Male

Reference Values},

ISSN = {0009-8981},

DOI = {10.1016/j.cca.2009.03.057},

year = {2009},

type = {Journal Article}

}

@article{NSE,

author = {Farrell, C. L. and Nguyen, L.},

title = {Indirect Reference Intervals: Harnessing the Power of Stored Laboratory Data},

journal = {Clin Biochem Rev},

volume = {40},

number = {2},

pages = {99-111},

note = {Farrell, Christopher-John L

Nguyen, Lan

Journal Article

Review

Australia

Clin Biochem Rev. 2019 May;40(2):99-111. doi: 10.33176/AACB-19-00022.},

abstract = {Reference intervals are relied upon by clinicians when interpreting their patients' test results. Therefore, laboratorians directly contribute to patient care when they report accurate reference intervals. The traditional approach to establishing reference intervals is to perform a study on healthy volunteers. However, the practical aspects of the staff time and cost required to perform these studies make this approach difficult for clinical laboratories to routinely use. Indirect methods for deriving reference intervals, which utilise patient results stored in the laboratory's database, provide an alternative approach that is quick and inexpensive to perform. Additionally, because large amounts of patient data can be used, the approach can provide more detailed reference interval information when multiple partitions are required, such as with different age-groups. However, if the indirect approach is to be used to derive accurate reference intervals, several considerations need to be addressed. The laboratorian must assess whether the assay and patient population were stable over the study period, whether data 'clean-up' steps should be used prior to data analysis and, often, how the distribution of values from healthy individuals should be modelled. The assumptions and potential pitfalls of the particular indirect technique chosen for data analysis also need to be considered. A comprehensive understanding of all aspects of the indirect approach to establishing reference intervals allows the laboratorian to harness the power of the data stored in their laboratory database and ensure the reference intervals they report are accurate.},

ISSN = {0159-8090 (Print)

0159-8090},

DOI = {10.33176/aacb-19-00022},

year = {2019},

type = {Journal Article}

}

@article{NSE,

author = {Ge lili, Yng Junmei, Li Jinxiang.},

title = {Establishment of reference interval of serum neuron-specific enolase of children in zhengzhou area},

journal = {Chongqing Medicine},

volume = {45},

number = {31},

pages = {4413-5},

abstract = {目的建立郑州地区0~14岁健康儿童血清神经元特异性烯醇化酶(NSE)的参考区间。方法检测1 001名0~14岁健康儿童血清NSE。将研究对象按性别和年龄分组(<1个月组、1个月至3岁组、>3岁组),对各项参数进行统计学分析,得出各年龄组儿童血清NSE的参考区间。结果 0~14岁儿童不同性别间血清NSE水平差异无统计学意义(P>0.05);不同年龄组间血清NSE水平差异有统计学意义(P<0.05)。3个年龄组的参考区间分别是0~32.13mg/L(<1月组),0~23.26mg/L(1个月至3岁组)和0~21.47mg/L(>3岁组)。结论儿童各年龄组的NSE参考区间具有明显差异。建立不同年龄段的NSE参考区间,对于临床疾病的诊断和评估具有重要意义。},

keywords = {神经元特异性烯醇化酶

参考值

儿童

郑州},

ISSN = {1671-8348},

DOI = {10. 3969／j. issn. 1671—8348. 2016. 31. 032},

url = {https://kns.cnki.net/KCMS/detail/detail.aspx?dbcode=CJFQ&dbname=CJFDLAST2017&filename=CQYX201631032&v=MTQ2NThSOGVYMUx1eFlTN0RoMVQzcVRyV00xRnJDVVI3cWZaT2R2RnkzaFY3M0FKanpTZHJHNEg5ZlBybzlHWm8=},

year = {2016},

type = {Journal Article}

}

@article{NSE,

author = {Horn, P. S. and Feng, L. and Li, Y. and Pesce, A. J.},

title = {Effect of outliers and nonhealthy individuals on reference interval estimation},

journal = {Clin Chem},

volume = {47},

number = {12},

pages = {2137-45},

note = {Horn, P S

Feng, L

Li, Y

Pesce, A J

eng

England

2001/11/24 10:00

Clin Chem. 2001 Dec;47(12):2137-45.},

abstract = {BACKGROUND: Improvement in reference interval estimation using a new outlier detection technique, even with a physician-determined healthy sample, is examined. The effect of including physician-determined nonhealthy individuals in the sample is evaluated. METHODS: Traditional data transformation coupled with robust and exploratory outlier detection methodology were used in conjunction with various reference interval determination techniques. A simulation study was used to examine the effects of outliers on known reference intervals. Physician-defined healthy groups with and without nonhealthy individuals were compared on real data. RESULTS: With 5% outliers in simulated samples, the described outlier detection techniques had narrower reference intervals. Application of the technique to real data provided reference intervals that were, on average, 10% narrower than those obtained when outlier detection was not used. Only 1.6% of the samples were identified as outliers and removed from reference interval determination in both the healthy and combined samples. CONCLUSIONS: Even in healthy samples, outliers may exist. Combining traditional and robust statistical techniques provide a good method of identifying outliers in a reference interval setting. Laboratories in general do not have a well-defined healthy group from which to compute reference intervals. The effect of nonhealthy individuals in the computation increases reference interval width by approximately 10%. However, there is a large deviation among analytes.},

keywords = {Disease

*Health Status

Humans

Normal Distribution

Reference Values},

ISSN = {0009-9147 (Print)

0009-9147 (Linking)},

year = {2001},

type = {Journal Article}

}

@article{NSE,

author = {Huang, L. and Zhou, J. G. and Yao, W. X. and Tian, X. and Lv, S. P. and Zhang, T. Y. and Jin, S. H. and Bai, Y. J. and Ma, H.},

title = {Systematic review and meta-analysis of the efficacy of serum neuron-specific enolase for early small cell lung cancer screening},

journal = {Oncotarget},

volume = {8},

number = {38},

pages = {64358-64372},

note = {1949-2553

Huang, Lang

Zhou, Jian-Guo

Yao, Wen-Xiu

Tian, Xu

Lv, Shui-Ping

Zhang, Ting-You

Jin, Shu-Han

Bai, Yu-Ju

Ma, Hu

Journal Article

United States

Oncotarget. 2017 May 11;8(38):64358-64372. doi: 10.18632/oncotarget.17825. eCollection 2017 Sep 8.},

abstract = {We performed a pooled analysis of the efficacy of serum neuron-specific enolase (NSE) levels for early detection of small cell lung cancer (SCLC) in patients with benign lung diseases and healthy individuals. Comprehensive searches of several databases through September 2016 were conducted. The quality of the included studies was assessed using the Quality Assessment of Diagnostic Accuracy Studies (QUADAS-2) tool. Ultimately, 33 studies containing 9546 samples were included in the review. Pooled sensitivity of NSE for detecting SCLC was 0.688 (95%CI: 0.627-0.743), specificity was 0.921 (95%CI: 0.890-0.944), positive likelihood ratio was 8.744 (95%CI: 6.308-12.121), negative likelihood ratio was 0.339 (95%CI: 0.283- 0.405), diagnostic odds ratio was 25.827 (95%CI: 17.490- 38.136) and area under the curve was 0.88 (95%CI: 0.85- 0.91). Meta-regression indicated that study region was a source of heterogeneity in the sensitivity and joint models, while cut-off level was a source in the joint model. Subgroup analysis showed that enzyme linked immunosorbent assays had the highest sensitivity and radioimmunoassay assays had the highest specificity. The diagnostic performance was better in Europe [sensitivity: 0.740 (95%CI: 0.676-0.795), specificity: 0.932 (95%CI: 0.904-0.953)] than in Asia [sensitivity: 0.590 (95%CI: 0.496- 0.678), specificity: 0.901 (95%CI: 0.819-0.948)]. In Europe, 25 ng/ml is likely the most suitable NSE cut-off level. NSE thus has high diagnostic efficacy when screening for SCLC, though the efficacy differs depending on study region, assay method and cut-off level. In the clinic, NSE measurements should be considered along with clinical symptoms, image results and histopathology.},

ISSN = {1949-2553},

DOI = {10.18632/oncotarget.17825},

year = {2017},

type = {Journal Article}

}

@article{NSE,

author = {Johansen, M. B. and Christensen, P. A.},

title = {A simple transformation independent method for outlier definition},

journal = {Clin Chem Lab Med},

volume = {56},

number = {9},

pages = {1524-1532},

note = {1437-4331

Johansen, Martin Berg

Christensen, Peter Astrup

Journal Article

Germany

Clin Chem Lab Med. 2018 Aug 28;56(9):1524-1532. doi: 10.1515/cclm-2018-0025.},

abstract = {BACKGROUND: Definition and elimination of outliers is a key element for medical laboratories establishing or verifying reference intervals (RIs). Especially as inclusion of just a few outlying observations may seriously affect the determination of the reference limits. Many methods have been developed for definition of outliers. Several of these methods are developed for the normal distribution and often data require transformation before outlier elimination. METHODS: We have developed a non-parametric transformation independent outlier definition. The new method relies on drawing reproducible histograms. This is done by using defined bin sizes above and below the median. The method is compared to the method recommended by CLSI/IFCC, which uses Box-Cox transformation (BCT) and Tukey's fences for outlier definition. The comparison is done on eight simulated distributions and an indirect clinical datasets. RESULTS: The comparison on simulated distributions shows that without outliers added the recommended method in general defines fewer outliers. However, when outliers are added on one side the proposed method often produces better results. With outliers on both sides the methods are equally good. Furthermore, it is found that the presence of outliers affects the BCT, and subsequently affects the determined limits of current recommended methods. This is especially seen in skewed distributions. The proposed outlier definition reproduced current RI limits on clinical data containing outliers. CONCLUSIONS: We find our simple transformation independent outlier detection method as good as or better than the currently recommended methods.},

keywords = {Adult

Blood Chemical Analysis/standards

Female

Humans

Laboratories, Hospital

Male

Reference Values

*Statistics, Nonparametric},

ISSN = {1434-6621},

DOI = {10.1515/cclm-2018-0025},

year = {2018},

type = {Journal Article}

}

@article{NSE,

author = {Katayev, A. and Balciza, C. and Seccombe, D. W.},

title = {Establishing reference intervals for clinical laboratory test results: is there a better way?},

journal = {Am J Clin Pathol},

volume = {133},

number = {2},

pages = {180-6},

note = {1943-7722

Katayev, Alex

Balciza, Claudiu

Seccombe, David W

Evaluation Studies

Journal Article

England

Am J Clin Pathol. 2010 Feb;133(2):180-6. doi: 10.1309/AJCPN5BMTSF1CDYP.},

abstract = {Reference intervals are essential for clinical laboratory test interpretation and patient care. Methods for estimating them are expensive, difficult to perform, often inaccurate, and nonreproducible. A computerized indirect Hoffmann method was studied for accuracy and reproducibility. The study used data collected retrospectively for 5 analytes without exclusions and filtering from a nationwide chain of clinical reference laboratories in the United States. The accuracy was assessed by the comparability of reference intervals as calculated by the new method with published peer-reviewed studies, and reproducibility was assessed by the comparability of 2 sets of reference intervals derived from 2 different data sets. There was no statistically significant difference between the calculated and published reference intervals or between the 2 sets of intervals that were derived from different data sets. A computerized Hoffmann method for indirect estimation of reference intervals using stored test results is proved to be accurate and reproducible.},

keywords = {Clinical Laboratory Techniques/*methods/statistics & numerical data

Humans

*Reference Values

Reproducibility of Results

Software},

ISSN = {0002-9173},

DOI = {10.1309/ajcpn5bmtsf1cdyp},

year = {2010},

type = {Journal Article}

}

@article{NSE,

author = {Lu Meihong, Ju Shaoqing, Cong Hui, Yang Shumei, Zhu Wencai.},

title = {Investigate of serum NSE reference range of health people in Nantong with Electrochemiluminescence},

journal = {Journal of Modern Laboratory Medicine},

volume = {33},

number = {4},

pages = {59-62},

abstract = {目的调查和建立南通地区表观健康人群神经元特异性烯醇化酶(neuron-specific enolase,NSE)的参考值区间。方法选取2017年1月~2018年1月南通大学附属医院体检中心3 874例健康体检者作为研究对象,其中男性2 767例,女性1 107例,通过问卷调查和体格检查排除神经系统、血液病、炎症、肿瘤等疾病,采用罗氏cobase 411全自动电化学发光仪检测NSE水平。根据性别、年龄将受检者分组(男女各7组:<30岁,30~39岁,40~49岁,50~59岁,60~69岁,70~79岁,>80岁),对结果进行统计学分析,建立相应的参考区间。分析性别、年龄分组后的组间差异是否有统计学意义;将获得的参考区间与试剂盒提供的生物参考区间进行比较,并进行参考区间的验证。结果 NSE水平呈正态分布,男性血清NSE水平(15.79±3.52ng/ml)明显高于女性(13.79±3.04ng/ml),差异有统计学意义(t=3.102,P=0.037)。但独立样本t检验进行各年龄组间两两比较差异无统计学意义(P>0.05)。采用x~-±1.96s确定参考区间,NSE参考区间为8.47~21.07ng/ml,男性为8.72~21.36ng/ml,女性为8.06~20.10ng/ml,对该研究建立的NSE参考区间进行验证符合标准。结论初步建立了适合该地区表观健康人群的血清NSE参考区间。NSE的参考区间有性别差异,根据不同性别建立的参考区间,可为疾病的诊断和治疗提供准确的依据.},

keywords = {神经元特异性烯醇化酶

年龄

性别

参考区间},

ISSN = {1671-7414},

DOI = {10.3969/j.issn.1671-7414.2018.04.015},

url = {https://kns.cnki.net/KCMS/detail/detail.aspx?dbcode=CJFQ&dbname=CJFDLAST2018&filename=SXYN201804015&v=MjkzMjRSN3FmWk9kdkZ5emtXci9QTmpYU1lMRzRIOW5NcTQ5RVlZUjhlWDFMdXhZUzdEaDFUM3FUcldNMUZyQ1U=},

year = {2018},

type = {Journal Article}

}

@article{NSE,

author = {Ozarda, Y.},

title = {Reference intervals: current status, recent developments and future considerations},

journal = {Biochem Med (Zagreb)},

volume = {26},

number = {1},

pages = {5-16},

note = {Ozarda, Yesim

Journal Article

Review

Croatia

Biochem Med (Zagreb). 2016;26(1):5-16. doi: 10.11613/BM.2016.001.},

abstract = {Reliable and accurate reference intervals (RIs) for laboratory analyses are an integral part of the process of correct interpretation of clinical laboratory test results. RIs given in laboratory reports have an important role in aiding the clinician in interpreting test results in reference to values for healthy populations. Since the 1980s, the International Federation of Clinical Chemistry (IFCC) has been proactive in establishing recommendations to clarify the true significance of the term 'RIs, to select the appropriate reference population and statistically analyse the data. The C28-A3 guideline published by the Clinical and Laboratory Standards Institute (CLSI) and IFCC is still the most widely-used source of reference in this area. In recent years, protocols additional to the Guideline have been published by the IFCC, Committee on Reference Intervals and Decision Limits (C-RIDL), including all details of multicenter studies on RIs to meet the requirements in this area. Multicentric RIs studies are the most important development in the area of RIs. Recently, the C-RIDL has performed many multicentric studies to obtain common RIs. Confusion of RIs and clinical decision limits (CDLs) remains an issue and pediatric and geriatric age groups are a significant problem. For future studies of RIs, the genetic effect would seem to be the most challenging area. The aim of the review is to present the current theory and practice of RIs, with special emphasis given to multicenter RIs studies, RIs studies for pediatric and geriatric age groups, clinical decision limits and partitioning by genetic effects on RIs.},

keywords = {Aged

Chemistry, Clinical/standards/statistics & numerical data

Child

Clinical Laboratory Techniques/*standards/*statistics & numerical data

Diagnostic Tests, Routine/standards/statistics & numerical data

Geriatrics/methods/standards/statistics & numerical data

*Guidelines as Topic

Humans

Pediatrics/methods/standards/statistics & numerical data

Reference Values},

ISSN = {1330-0962 (Print)

1330-0962},

DOI = {10.11613/bm.2016.001},

year = {2016},

type = {Journal Article}

}

@article{NSE,

author = {Park, S. H. and Hwang, S. K.},

title = {Prognostic Value of Serum Levels of S100 Calcium-Binding Protein B, Neuron-Specific Enolase, and Interleukin-6 in Pediatric Patients with Traumatic Brain Injury},

journal = {World Neurosurg},

volume = {118},

pages = {e534-e542},

note = {Park, Seong-Hyun

Hwang, Sung-Kyoo

eng

2018/09/28 06:00

World Neurosurg. 2018 Oct;118:e534-e542. doi: 10.1016/j.wneu.2018.06.234. Epub 2018 Jul 6.},

abstract = {OBJECTIVE: To analyze serum levels of S100 calcium-binding protein B (S100B), neuron-specific enolase (NSE), and interleukin (IL)-6 in pediatric patients with traumatic brain injury (TBI) and to assess their relationship with clinical outcome. METHODS: To measure biomarkers, peripheral venous blood was collected within 6 hours and 1 week after TBI. Initial Glasgow Coma Scale (GCS) scores and Glasgow Outcome Scale scores 6 months after the trauma were used to evaluate clinical outcome. RESULTS: Median serum levels of S100B (178.12 pg/mL), NSE (16.54 ng/mL), and IL-6 (15.48 pg/mL) at admission decreased significantly 1 week after TBI to 40.86 pg/mL, 5.85 ng/mL, and 8.63 pg/mL. In the group with poor GCS scores, serum S100B and NSE levels both at admission and 1 week after TBI were significantly higher than levels in the group with good GCS scores. Serum S100B and NSE levels 1 week after injury in patients with unfavorable 6-month outcomes were significantly higher than levels 1 week after injury in patients with favorable outcomes. CONCLUSIONS: Serum levels of S100B, NSE, and IL-6 decreased 1 week after injury. Serum levels of S100B and NSE at admission were related to initial GCS scores, and these levels 1 week after TBI were related to 6-month Glasgow Outcome Scale scores. Thus, serial measurements of serum S100B and NSE, but not IL-6, may help assess brain damage and clinical outcome of pediatric patients with TBI.},

keywords = {Adolescent

Biomarkers/blood

Brain Injuries, Traumatic/*blood/*diagnosis

Child

Child, Preschool

Female

Glasgow Coma Scale/trends

Humans

Interleukin-6/*blood

Male

Phosphopyruvate Hydratase/*blood

Prognosis

Prospective Studies

S100 Calcium Binding Protein beta Subunit/*blood},

ISSN = {1878-8769 (Electronic)

1878-8750 (Linking)},

DOI = {10.1016/j.wneu.2018.06.234},

url = {http://www.ncbi.nlm.nih.gov/pubmed/30257306},

year = {2018},

type = {Journal Article}

}

@article{NSE,

author = {Planche, V. and Brochet, C. and Bakkouch, A. and Bernard, M.},

title = {[Importance of hemolysis on neuron-specific enolase measurement]},

journal = {Ann Biol Clin (Paris)},

volume = {68},

number = {2},

pages = {239-42},

note = {Planche, Virginie

Brochet, Christine

Bakkouch, Asma

Bernard, Maguy

English Abstract

Journal Article

France

Ann Biol Clin (Paris). 2010 Mar-Apr;68(2):239-42. doi: 10.1684/abc.2010.0422.},

abstract = {The aim of this work is to establish a pre-analytical approach suitable for the neuron-specific enolase (NSE) measurement. This enzyme which is synthesized by neurons and neuroendocrine cells, is a marker useful for the diagnosis and the monitoring of patients with neuroendocrine tumors (neuroblastoma, small cell lung cancer) and during stroke to assess neuronal damage. This NSE measurement is very sensitive to hemolysis due to the abundance of the enzyme in red blood cells. Two methods of evaluation of hemolysis have been compared: the determination of free haemoglobin (Hb) by spectrophotometry and the indirect measurement of an hemolytic index with a multiparameter analyzer, the Modular (Roche Diagnostics). The correlation between these 2 methods on 42 samples is very satisfactory: Y (free Hb) = 12.337 X (index) + 31.743 r = 0.997. The NSE assay is based on TRACE (Time Resolved Amplified Cryptate Emission) technology, on a Kryptor (BRAHMS). The influence of hemolysis on the determination of NSE was confirmed by overloading with hemoglobin (hemolysate) 3 pools of serum with NSE concentrations close to the threeshold decision. The determination of NSE shows an increase in concentration parallely to the hemolytic index (about 150% for an hemolytic index of 10). Consequently, in our laboratory the NSE determination is realized only for samples presenting an hemolytic index < or = 10, this allowing a good monitoring of kinetics of this marker.},

keywords = {Biomarkers, Tumor/blood

Hemoglobins/metabolism

*Hemolysis

Humans

Lung Neoplasms/blood/enzymology/pathology

Neuroblastoma/blood/enzymology/pathology

Neurons/enzymology/pathology

Phosphopyruvate Hydratase/biosynthesis/*blood

Small Cell Lung Carcinoma/blood/enzymology/pathology

Spectrophotometry/methods},

ISSN = {0003-3898 (Print)

0003-3898},

DOI = {10.1684/abc.2010.0422},

year = {2010},

type = {Journal Article}

}

@article{NSE,

author = {Thelin, E. P. and Jeppsson, E. and Frostell, A. and Svensson, M. and Mondello, S. and Bellander, B. M. and Nelson, D. W.},

title = {Utility of neuron-specific enolase in traumatic brain injury; relations to S100B levels, outcome, and extracranial injury severity},

journal = {Crit Care},

volume = {20},

pages = {285},

note = {Thelin, Eric Peter

Jeppsson, Emma

Frostell, Arvid

Svensson, Mikael

Mondello, Stefania

Bellander, Bo-Michael

Nelson, David W

eng

England

London, England

2016/09/09 06:00

Crit Care. 2016 Sep 8;20:285. doi: 10.1186/s13054-016-1450-y.},

abstract = {BACKGROUND: In order to improve assessment and outcome prediction in patients suffering from traumatic brain injury (TBI), cerebral protein levels in serum have been suggested as biomarkers of injury. However, despite much investigation, biomarkers have yet to reach broad clinical utility in TBI. This study is a 9-year follow-up and clinical experience of the two most studied proteins, neuron-specific enolase (NSE) and S100B, in a neuro-intensive care TBI population. Our aims were to investigate to what extent NSE and S100B, independently and in combination, could predict outcome, assess injury severity, and to investigate if the biomarker levels were influenced by extracranial factors. METHODS: All patients treated at the neuro-intensive care unit at Karolinska University Hospital, Stockholm, Sweden between 2005 and 2013 with at least three measurements of serum S100B and NSE (sampled twice daily) were retrospectively included. In total, 417 patients fulfilled the criteria. Parameters were extracted from the computerized hospital charts. Glasgow Outcome Score (GOS) was used to assess long-term functional outcome. Univariate, and multivariate, regression models toward outcome and what explained the high levels of the biomarkers were performed. Nagelkerke's pseudo-R(2) was used to illustrate the explained variance of the different models. A sliding window assessed biomarker correlation to outcome and multitrauma over time. RESULTS: S100B was found a better predictor of outcome as compared to NSE (area under the curve (AUC) samples, the first 48 hours had Nagelkerke's pseudo-R(2) values of 0.132 and 0.038, respectively), where the information content of S100B peaks at approximately 1 day after trauma. In contrast, although both biomarkers were independently correlated to outcome, NSE had limited additional predictive capabilities in the presence of S100B in multivariate models, due to covariance between the two biomarkers (correlation coefficient 0.673 for AUC 48 hours). Moreover, NSE was to a greater extent correlated to multitrauma the first 48 hours following injury, whereas the effect of extracerebral trauma on S100B levels appears limited to the first 12 hours. CONCLUSIONS: While both biomarkers are independently correlated to long-term functional outcome, S100B is found a more accurate outcome predictor and possibly a more clinically useful biomarker than NSE for TBI patients.},

keywords = {Adult

Biomarkers/analysis/blood

Brain Injuries, Traumatic/epidemiology

Female

Glasgow Coma Scale/statistics & numerical data

Glasgow Outcome Scale/statistics & numerical data

Humans

Logistic Models

Male

Middle Aged

*Patient Outcome Assessment

Phosphopyruvate Hydratase/*analysis/blood

Prognosis

Retrospective Studies

S100 Calcium Binding Protein beta Subunit/*analysis/blood

Sweden/epidemiology},

ISSN = {1466-609X (Electronic)

1364-8535 (Linking)},

DOI = {10.1186/s13054-016-1450-y},

url = {http://www.ncbi.nlm.nih.gov/pubmed/27604350},

year = {2016},

type = {Journal Article}

}

@article{NSE,

author = {van Adrichem, R. C. and Kamp, K. and Vandamme, T. and Peeters, M. and Feelders, R. A. and de Herder, W. W.},

title = {Serum neuron-specific enolase level is an independent predictor of overall survival in patients with gastroenteropancreatic neuroendocrine tumors},

journal = {Ann Oncol},

volume = {27},

number = {4},

pages = {746-7},

note = {1569-8041

van Adrichem, R C S

Kamp, K

Vandamme, T

Peeters, M

Feelders, R A

de Herder, W W

Letter

England

Ann Oncol. 2016 Apr;27(4):746-7. doi: 10.1093/annonc/mdv626. Epub 2015 Dec 27.},

keywords = {Adult

Aged

Disease-Free Survival

Female

Humans

Intestinal Neoplasms/*blood/pathology

Male

Middle Aged

Neuroendocrine Tumors/*blood/pathology

Neurons/enzymology/pathology

Pancreatic Neoplasms/*blood/pathology

Phosphopyruvate Hydratase/*blood

*Prognosis

Stomach Neoplasms/*blood/pathology},

ISSN = {0923-7534},

DOI = {10.1093/annonc/mdv626},

year = {2016},

type = {Journal Article}

}

@article{NSE,

author = {Woo, H. Y. and Kim, Y. J. and Park, H.},

title = {[Establishment of reference intervals of tumor markers in Korean adults]},

journal = {Korean J Lab Med},

volume = {28},

number = {3},

pages = {179-84},

note = {Woo, Hee-Yeon

Kim, Young Jae

Park, Hyosoon

kor

English Abstract

Korea (South)

2008/07/03 09:00

Korean J Lab Med. 2008 Jun;28(3):179-84. doi: 10.3343/kjlm.2008.28.3.179.},

abstract = {BACKGROUND: The sensitivity and specificity of tumor markers for detecting cancer could be significantly changed by the reference intervals of tumor markers. We established reference intervals of tumor markers in Korean adults and evaluated its importance, since the reference intervals recommended by the manufacturers were determined in the Caucasian population and have sometimes been adopted without verification. METHODS: We established the reference intervals of alpha fetoprotein (AFP), carcinoembryonic antigen (CEA), cancer antigen (CA)125, carbohydrate antigen (CA)19-9, total prostate specific antigen (TPSA), cytokeratin fragment (Cyfra)21-1, and neuron specific enolase (NSE) according to the CLSI guideline in a maximum number of 1,364 healthy adults aged 20-60 yrs who visited a health promotion center from January to February 2007. RESULTS: Reference intervals of all tumor markers except for AFP were not in agreement with those recommended by the manufacturers. Reference intervals of CEA, TPSA, CA19-9, CA125, and Cyfra21-1 were age dependent. The mean reference values of NSE, CA125, and CEA were statistically different according to gender (11.72 vs 10.78 ng/mL), menopause status (18.89 vs 12.62 U/mL), and smoking status (2.60 vs 2.12 vs 1.80 ng/mL for smokers, past smokers, and non-smokers, respectively),respectively. CONCLUSIONS: With the verification and establishment of reference intervals of tumor markers in a Korean local population, we found the reference intervals significantly different by either age, gender, smoking or menopause status.},

keywords = {Adult

Age Factors

Biomarkers, Tumor/*standards

Carcinoembryonic Antigen/analysis

Female

Humans

Korea

Male

Menopause

Middle Aged

Phosphopyruvate Hydratase/analysis

Prostate-Specific Antigen/analysis

Reagent Kits, Diagnostic

Reference Values

Sex Factors

Smoking

Surveys and Questionnaires

alpha-Fetoproteins/analysis},

ISSN = {1598-6535 (Print)

1598-6535 (Linking)},

DOI = {10.3343/kjlm.2008.28.3.179},

url = {http://www.ncbi.nlm.nih.gov/pubmed/18594168},

year = {2008},

type = {Journal Article}

}

@article{NSE,

author = {Yang, J. and Tang, A. and Ma, J. and Sun, X. and Ming, L.},

title = {The reference intervals for CA125, CA15-3, CA19-9, CA72-4, AFP, CEA, NSE and CYFRA21-1},

journal = {Scand J Clin Lab Invest},

volume = {79},

number = {1-2},

pages = {71-74},

note = {1502-7686

Yang, Jingjing

Tang, Aiguo

Ma, Junfen

Sun, Xiaoxu

Ming, Liang

Journal Article

England

Scand J Clin Lab Invest. 2019 Feb - Apr;79(1-2):71-74. doi: 10.1080/00365513.2018.1555855. Epub 2019 Feb 6.},

abstract = {Tumor markers are noninvasive diagnostic tools for cancer. Their abnormal expression often occurs earlier than clinical symptoms or other detection signals. Appropriate reference intervals (RIs) of tumor markers are important for health evaluation, cancer diagnosis, therapy monitoring and prognosis assessment. In this study, we aimed to establish the RIs of cancer antigen 125 (CA125), CA15-3, CA19-9, CA72-4, alpha fetoprotein (AFP), carcino-embryonic antigen (CEA), neuron-specific enolase (NSE) and cytokeratin 19 fragment antigen 21-1 (CYFRA21-1) in apparently healthy Henan population. A total of 1705 apparently healthy participants (21-89 years) were recruited from five representative geographical regions in Henan province. Nonparametric 95th percentile intervals were used to define the RIs of CA125, CA15-3, CA19-9, CA72-4, AFP, CEA, NSE and CYFRA21-1. The test results of CA125, CA15-3, CA19-9, CA72-4, AFP, CEA, NSE and CYFRA21-1 can traceable to reference measurement procedures. The age- and gender-specific RIs of the tumor markers were established. We established age- and gender-specific RIs for CA125, CA15-3, CA19-9, CA72-4, AFP, CEA, NSE and CYFRA21-1. The newly established RIs should be more suitable for Henan population. It will be valuable for clinicians to make a medical diagnosis, therapeutic management decision and other physiological assessment.},

keywords = {Adult

Age Factors

Aged

Aged, 80 and over

Antigens, Neoplasm/blood/*genetics

Antigens, Tumor-Associated, Carbohydrate/blood/*genetics

Biomarkers, Tumor/blood/*genetics

CA-125 Antigen/blood/*genetics

CA-19-9 Antigen/blood/*genetics

Carcinoembryonic Antigen/blood/*genetics

China

Female

Healthy Volunteers

Humans

Keratin-19/blood/*genetics

Male

Middle Aged

Mucin-1/blood/*genetics

Phosphopyruvate Hydratase/blood/*genetics

Pregnancy

Reference Values

Sex Factors

alpha-Fetoproteins/*genetics/metabolism},

ISSN = {0036-5513},

DOI = {10.1080/00365513.2018.1555855},

year = {2019},

type = {Journal Article}

}

@article{NSE,

author = {Zhou, M. and Wang, Z. and Yao, Y. and Zhou, H. and Liu, M. and Sun, J.},

title = {Neuron-specific enolase and response to initial therapy are important prognostic factors in patients with small cell lung cancer},

journal = {Clin Transl Oncol},

volume = {19},

number = {7},

pages = {865-873},

note = {Zhou, M

Wang, Z

Yao, Y

Zhou, H

Liu, M

Sun, J

eng

Italy

2017/01/28 06:00

Clin Transl Oncol. 2017 Jul;19(7):865-873. doi: 10.1007/s12094-017-1617-2. Epub 2017 Jan 26.},

abstract = {PURPOSE: The prognostic factors for the survival of small cell lung cancer (SCLC) patients are still widely debated. The aim of this study was to identify the clinical features and prognostic factors in SCLC patients. METHODS: A retrospective study was conducted on SCLC patients who were treated in our hospital between July 2010 and July 2015. Comparison of overall survival (OS) was performed using the Kaplan-Meier method. Prognostic factors for OS were identified by multivariate Cox regression models. RESULTS: A total of 523 patients with complete data and ECOG 0-2 were enrolled in our study. A total of 383 patients (73.2%) were diagnosed with ES-SCLC (extensive-stage SCLC) and 140 patients (26.8%) were diagnosed with LS-SCLC (limited-stage SCLC). In all patients, early disease stage, good ECOG, normal neuron-specific enolase (NSE), thoracic radiotherapy, >/=4 cycles of chemotherapy, prophylactic cranial irradiation, good response to initial therapy were independent favorable prognostic factors for OS, along with gender, age, CEA and CA125. In LS-SCLC patients, normal NSE, normal CEA, good response to initial therapy and surgery were independent favorable prognostic factors for OS. In ES-SCLC patients, good ECOG, normal NSE, thoracic radiotherapy, >/=4 cycles of chemotherapy, prophylactic cranial irradiation and good response to initial therapy were independent favorable prognostic factors for OS. Remarkably, NSE and response to initial therapy were independent prognostic factors for OS in all SCLC patients, LS-SCLC patients and ES-SCLC patients. CONCLUSION: The normal NSE and good response to initial therapy predicted a better survival for SCLC patients, regardless of disease stage.},

keywords = {Adult

Aged

Aged, 80 and over

Antineoplastic Combined Chemotherapy Protocols/*therapeutic use

Biomarkers, Tumor/*metabolism

Chemoradiotherapy

*Cranial Irradiation

Female

Follow-Up Studies

Humans

Lung Neoplasms/metabolism/*pathology/therapy

Male

Middle Aged

Phosphopyruvate Hydratase/*metabolism

Prognosis

Retrospective Studies

Small Cell Lung Carcinoma/metabolism/*pathology/therapy

Survival Rate},

ISSN = {1699-3055 (Electronic)

1699-048X (Linking)},

DOI = {10.1007/s12094-017-1617-2},

url = {http://www.ncbi.nlm.nih.gov/pubmed/28127669},

year = {2017},

type = {Journal Article}

}
